# Three dimensional reconstructions of *Nummulites* tests reveal complex chamber shapes

**DOI:** 10.7717/peerj.1072

**Published:** 2015-07-02

**Authors:** Willem Renema, Laura Cotton

**Affiliations:** Naturalis Biodiversity Center, CR Leiden, The Netherlands

**Keywords:** Large benthic foraminifera, Character evaluation, Micropaleontology, Computed microtomography

## Abstract

Larger benthic foraminifera (LBF) are important and prolific carbonate producers both in modern and ancient shallow tropical seas. During the Paleogene the genus *Nummulites* was particularly abundant with a global distribution, leading it to be frequently used in biostratigraphy. However, their evolution is poorly understood as classification is Europe-centered and mostly based on external characters and equatorial thin sections. New occurrences from regions outside the northern Tethys which poorly fit in thus reference frame, show that a more rigid framework for the classification of *Nummulites* is needed. Here we apply micro computed-tomographical scanning, a tool that recently has become available, to visualise 3D chamber shape of *Nummulites djokdjokartae* and compare these to traditional morphometrical characters. We find that despite the regular shape in equatorial and axial thin section the irregular 3D chamber shape is not predicted by these sections. We argue that 3D reconstructions of *Nummulites* tests will be a great aid in improving our understanding of lineages within the genus *Nummulites*, and to elucidate its evolutionary and biogeographical history.

## Introduction

During the Eocene, 55–35 million years ago, larger benthic foraminifera (LBF) were one of the most important carbonate producers in shallow marine tropical conditions (Kiessling, Flügel & Golonka, 2003). The ranges of species of nummulitids, alveolinids, and orthophragminids form a basis for the shallow marine zonation (SBZ) for the Paleogene of the western Tethys (Serra-Kiel et al., 1998). The basis for the use of LBF for stratigraphy lies in the phylogenetic relationship between taxa. The genus *Nummulites* is particularly abundant in many Eocene deposits and contains over 500 described species (e.g., Schaub, 1981). However, differing prioritisation of characters, species concepts, and reference frameworks have resulted in a taxonomic maze. The most frequently used reference work is the revision of *Nummulites* by Schaub (1981), who classified the genus into lineages (which he called ‘phyla’) based on externally visible characters (e.g., shape of the septal trace, number, size, and distribution of pillars), and internal characters from equatorial thin sections (e.g., chamber length and septal shape). Within these lineages the species are separated by biometrical characteristics, primarily the size of the first chamber (proloculus) in the A-form, secondarily by ontogenetic increase of the whorl radius, both measured in equatorial thin sections. Within most lineages the species are ordered by increasing proloculus size, but also by the number of whorls, the diameter, and increasing size difference between the sexual and a-sexual generation. The youngest members of these lineages are the most distinct, but the ancestral members of the lineages are often difficult to separate. Many smaller, more simple shaped specimens cannot be placed in these lineages other than by geographical association. Furthermore, most of the material examined by Schaub (1981) is from southwest Europe, with few samples from central and eastern Europe. Since then, faunal descriptions from several regions have been published, for example from Oman (Racey, 1995), Israel (Schaub, 1995), but these have all worked within the Schaub reference framework, or used the same external characters to define species groups (but not lineages). The latter was especially done in areas with many species that do not fit with Schaub’s (1981) paper, such as India (Samanta, Bandopadhyay & Lahiri, 1990) among others. Recently, the paradigm that morphological change in LBF occurs at the same time and rate over large geographical distances has been questioned (Renema, 2015), highlighting the need for an updated reference framework in which also non-European occurrences fit.

Bottlenecks in our understanding of the evolution of *Nummulites* have arisen partially because the majority of characters used are derived from looking at specimens in equatorial thin section and external view only. Axial sections are not widely used. For example, in Schaub’s (1981) revision of western Tethyan *Nummulites* very few axial sections were figured. It was not until 1991 that Kleiber added the axial sections to most of Schaub’s species. Based on external observations, Adams (1988) emphasized that the shape of the septal filaments is an important character, and he provided a reference framework for categorising them. Racey (1992) assessed the degree of interdependence of morphological characters and concluded that internal characters evaluated in equatorial and axial thin section were the most important. However, contrary to Schaub (1981), he valued characters in axial thin sections as important as those in equatorial sections. The presence, distribution, and shape of pillars, the thickness and extent of alar prolongations, and the shape of the chamber lumen are very useful characters to place *Nummulites* in lineages (Kleiber, 1991; Racey, 1992), but their application has been limited since species identification requires equatorial sections.

The recent advance of high resolution computed tomography in paleontology has enabled the reconstruction of the three-dimensional morphology of foraminifera tests (Speijer et al., 2008; Briguglio, Metscher & Hohenegger, 2011; Görög et al., 2012). Here we present 3D models of *Nummulites* tests with the purpose of increasing our understanding of morphology and growth of complex *Nummulites* in order to assess whether two dimensional analyses are truly representative of the test and whether key morphological traits are being lost during this preparation technique. Using *N. djokdjokartae* as a model organism, we will demonstrate that chamber shape is not well represented in either equatorial or axial thin sections, but that it is an important character to understand *Nummulites* growth and, as a derivative, paleobiogeography, and evolution.

## Methods

Eocene *Nummulites djokdjokartae* were collected from four stratigraphic levels within the Nanggulan Formation at Kali Watupuruh, Nanggulan, Central Java. The Nanggulan Formation is a sequence of overall deepening upwards marine mudstones, sandstones, and conglomerates. At the top of the sampled interval the presence of abundant large *Discocyclina* indicates that this stratigraphic level correlates with the middle Bartonian Ta-Tb boundary (see Renema, 2007; Lunt, 2013). The samples are therefore considered to be early Bartonian (38–40 Ma) in age.

We scanned 74 tests of *Nummulites djokdjokartae* from four levels. For the scanning we used a Bruker/Skyscan 1172 at least 5–7 µm pixel size resolution. Settings used for scanning the specimens were 2,000 × 1,336 pixels, aluminum filter, 80 Kv, camera rotation step of 0.15 degrees, and an exposure time of 800–1,000 ms, depending on the size of the specimen. These specimens were used to characterise virtual equatorial thin sections comparable to those used in traditional descriptions of *Nummulites* populations. In these sections we measured proloculus size, number of whorls, number of chambers per whorl, whorl radius, chamber height, and proximal and distal angles of the septa.

Seven specimens were used to reconstruct the 3D chamber shape. To do this, the reconstructed image stack was imported into Avizo, in which we segmented all successive chamber cavities (chamber lumen and alar prolongations). We did this by first automatically filling all the openings in the test, followed by twice automatically growing and shrinking the volume to eliminated the small connections of the stolon system. Following this procedure the chambers were manually segmented and numbered.

**Figure 1:**
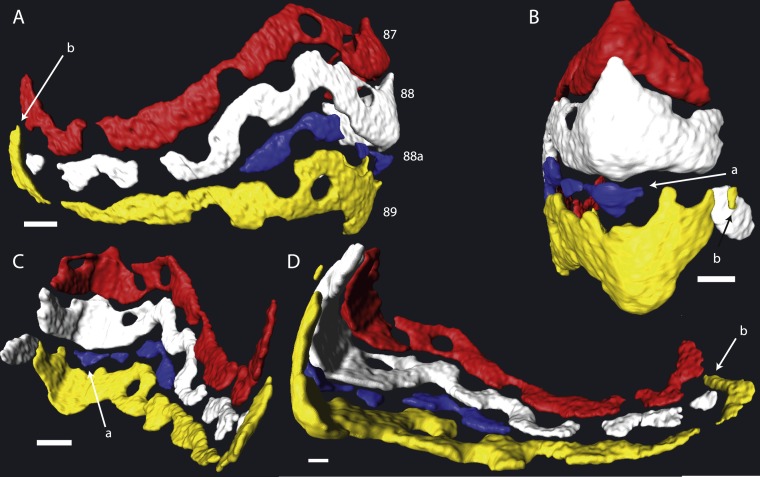
Detail of chambers 87–89 of specimen 06KW01_05 in four different perspectives. (A) Axial view. (B) Outside peripheral view. (C) Inside peripheral view. (D) Oblique inside view. (a) Small chamber with a single alar prolongation. (b) Superposition of alar prolongation over the alar prolongation of the previous whorl. Scale bar is 250 µm.

**Figure 2:**
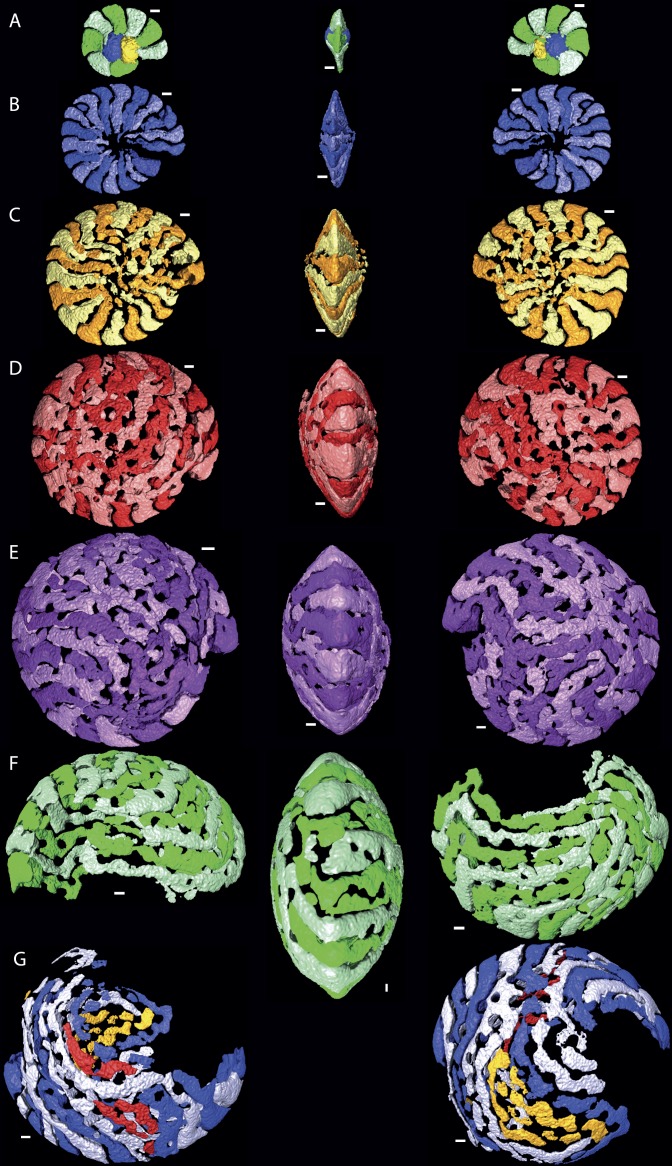
Chamber shape per whorl in specimen 06KW01_05. Note the increase in complexity of chamber shape. (A) whorl 1; (B) whorl 2; (C) whorl 3; (D) whorl 4; (E) whorl 5; (G) first half of whorl 6; (H) second half of whorl 6. In yellow and red that could not attributed to a chamber with certainty are indicated. Note that in whorl 6 the alar prolongations of half a whorl cover up to 3/4 of the test. Scale bar is 250 µm. 3D movies of this figure are in the SOM.

In most cases chamber shape is obvious and could be reconstructed by selecting the space within the chamber and allowing Avizo to automatically extrapolate the chamber. In rare occasions one of several problems could arise: (1) The chamber is very small, and consists mostly of an alar prolongation (Fig. 1, chamber 88a). Such a chamber was included when it appeared in the virtual equatorial section. (2) The chamber is highly asymmetrical, and has one long and one reduced alar prolongation (Fig. 1, chamber 89). Such a chamber was included when it appeared as a chamber in the virtual equatorial section. Otherwise it was included in the chamber to which it connected. (3) Especially in later whorls, chamber shapes could become complex, and the presence of pillars rendered them discontinuous. In most cases the connections between chambers were obvious, but in rare occasions the law of superposition was used: older chambers are overlain by younger chambers (Fig. 1B). In one specimen (06KW01_05) four lateral parts of chambers could not unambiguously be related to a growth increment with certainty (Fig. 2). Following segmentation the volume of each chambers were calculated using the material statistics option in Avizo 8.1.

## Results

### Morphology in 2D thin sections

No morphological differences were found between the four samples examined, hence all specimens are treated as coming from a single population. In equatorial sections chamber shape changed from almost square (width is similar to height) to elongated (width is up to 3 times longer than height). In nearly all specimens square chambers formed less than a whorl, resulting in a straight line in the whorl diagram (Fig. 4). However, based on total number of whorls and proloculus diameter two groups can be recognized, group A1 with a proloculus size of smaller than 400 µm, and more than 5 whorls, and group A2 with a proloculus larger than 400 µm and fewer than 5 whorls (Fig. 5). The marginal cord is placed in a slightly undulating plane perpendicular to the coiling. Especially in group A1 the outermost whorls are bent out of the plane of coiling. The general morphology in both groups includes several initial chambers that are as high as long, followed by 3–5 whorls with chambers that are up to 3 times longer than high (Figs. 6A and 6B). Overall this results in an increase in chamber length and a comparable number of chambers per whorl in whorls 3–5, despite the increase in radius (Fig. 7A). Chamber length is variable though, with occasional very narrow chambers or split septa (Figs. 6 and 7B). Proximal and distal angle of the septa is comparable in all specimens. The total number of chambers differ between the A1 and A2 group; specimens in A1 have 130–150 chambers and those in A2 90–110 chambers.

### Chamber shape in 3D

Despite the relatively regular shape in equatorial and axial thin section, chamber morphology becomes increasingly more complex in each whorl. Chamber shape is typical for *Nummulites* in the first one to three whorls, with alar prolongations extending to the central part of the test, and are symetrical on either side of the equatorial plane. In rare occasions small chambers without alar prolongations occur. In most cases these chambers are not recognisable in equatorial thin section other than by their very short lengths. In succesive whorls this shape becomes more irregular as the result of extending the alar prolongations. Simultaneously the alar prolongations become narrower and assymetrical. They form at an angle to the line from the septum to the central area of the test. In whorl three and four most alar prolongations are not interrupted. However in subsequent whorls alar prolongations are narrowed by pustules which are visible in successive chambers and lie in the chamber, not on the septal filaments. With increasing chamber number the length of the alar prolongations becomes longer, their shape more irregular, and the asymmetry over the equatorial plane increases. Usually succesive chambers have one long and one short alar prolongation on the same side of the test, followed by a few chambers where this pattern is mirrored. In the first 50 chambers this pattern is comparable between specimens from group A1 and A2. Specimens in group A1 have more chambers, and the chambers in the last whorl become increasingly more irregular and more dificult to reconstruct due to partitioning of the (long) alar prolongation. These alar prolongations curve around the central part of the test. The longest alar prolongations can make a 270 degree rotation (Fig. 2). The stacking of these thin alar prolongations results in the very regular axial section which almost has the appearance of lateral chamberlets as observed in *Spiroclypeus*.

## Discussion

Our results provide new insights into the evaluation of morphological characters in two versus three dimensions. We argue that when only using external views and oriented thin sections characters can be misinterpreted and that understanding the 3D structure is necessary to enable to a better understanding of *Nummulites* evolution, and as a consequence their use in biostratigraphy.

### Primary, secondary, and tertiary septal filaments

Based solely on the external morphology of *Nummulites* tests, Adams (1988) categorised the septal filament traces into three groups. Primary septal filaments arise directly from the septa. Secondary filaments are the distal walls of chamberlets and develop in reticulate and sub-reticulate *Nummulites*. Tertiary filaments originate as spiral ridges or spurs from the primary or secondary septal filaments. However, he does not provide a model of how these structures form during growth. Foraminifera grow by adding chambers to the apertural face of the test. However, exactly how this method of incremental growth results in the different categories of septal filaments is not explained by Adams (1988). Our study shows that in *Nummulites djokdjokartae*, which is closely related to *N. britannicus* used in Adams’ study (Renema, Racey & Lunt, 2003), all three categories of septal filaments are present. Our data show, however, that all three have the same origin, and are the traces of the primary septal filaments. The apparent presence of secondary septal traces, resulting in branching structures, is the result of superposition of alar prolongations from within the same whorl. This superposition is not apparent in traditional growth models of *Nummulites*, and is the result of the irregular chamber shape with alar prolongations extending in variable lengths over the external surface of the test. These vary from being almost straight and extending to the polar region, to extending around the polar region for almost two-thirds of the shell. Superposition of these two chamber types results in apparent branching patterns in the traces of the septal filaments. In extreme cases chambers run almost parallel to the marginal cord, and form spiral ridges or the tertiary septal filaments as defined by Adams (1988).

### Two dimensional sections overemphasise the regularity of nummulitid tests

**Figure 3:**
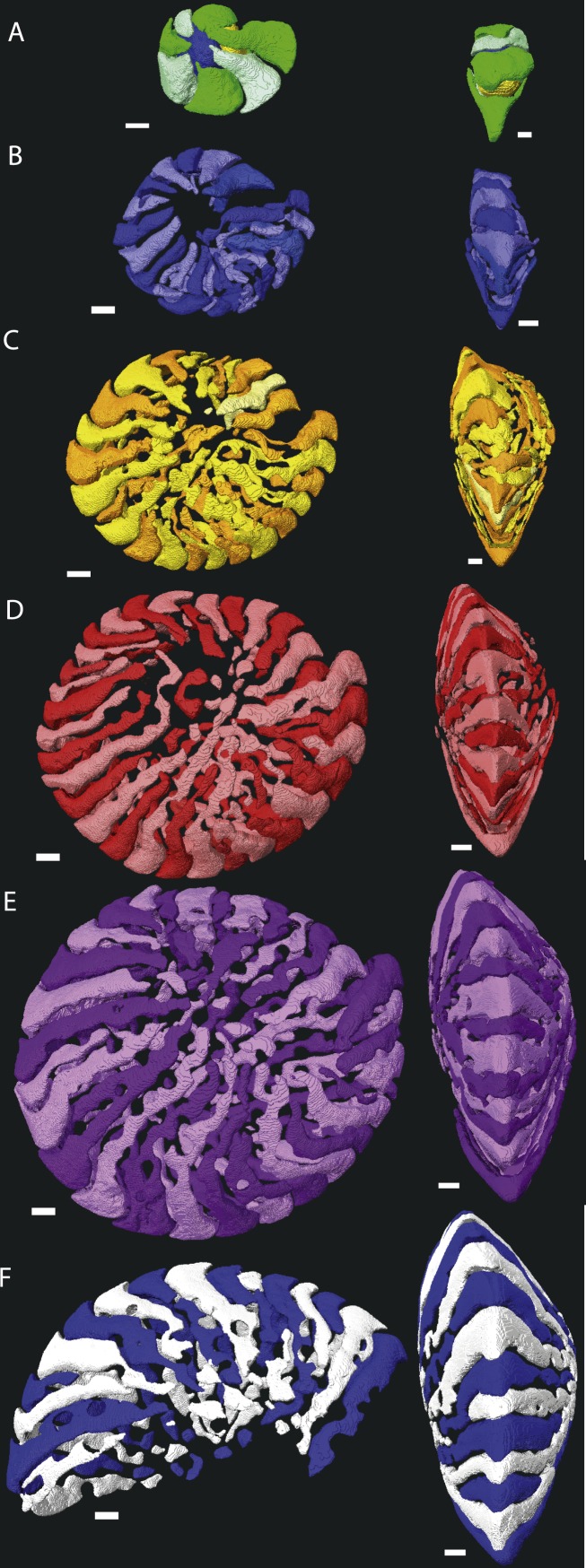
Chamber shape per whorl in specimen 06KW01_19. Note the difference with specimen 06KW01_05 (Fig. 2). (A) whorl 1; (B) whorl 2; (C) whorl 3; (D) whorl 4; (E) whorl 5; (G) first half of whorl 6. 3D movies of this figure are in the SOM.

Macrospheric specimens of *Nummulites djokdjokartae* have a very regular appearance in both equatorial horizontal and axial thin section. Equatorial section there are 4–6 whorls with chambers with almost straight septa and chamber shape which changes from being higher than long in the initial whorl to longer than high in the later whorls. In axial section there are stacks of alar prolongations of very similar height visible throughout the test, including the polar region. In traditional growth models of *Nummulites* with the chambers converging in the polar region, it is expected that alar prolongations narrow towards the center and often a polar pillar is present. Although the regularity of the stack of alar prolongations gives the impression that all chambers have long alar prolongations aligned next to each other, when the 3D reconstructions of the chambers are examined, it becomes clear that frequently alar prolongations of non-successive chambers are aligned next to each other in the central part of the test, and that sometimes the same alar prolongation transverses an axial plane multiple times (Figs. 2 and 3). In axial thin section the test appears symmetrical, but other than the first ten or so regular chambers, all chambers are asymmetrical in the equatorial plane. This does not conform the description of the genus *Nummulites*, which includes involute, biconvex, planispiral coiling (Loeblich & Tappan, 1988; Schaub, 1981). Depending on the distribution of this character within Schaub’s phyla, an emendation of the description of *Nummulites* might be needed.

**Figure 4:**
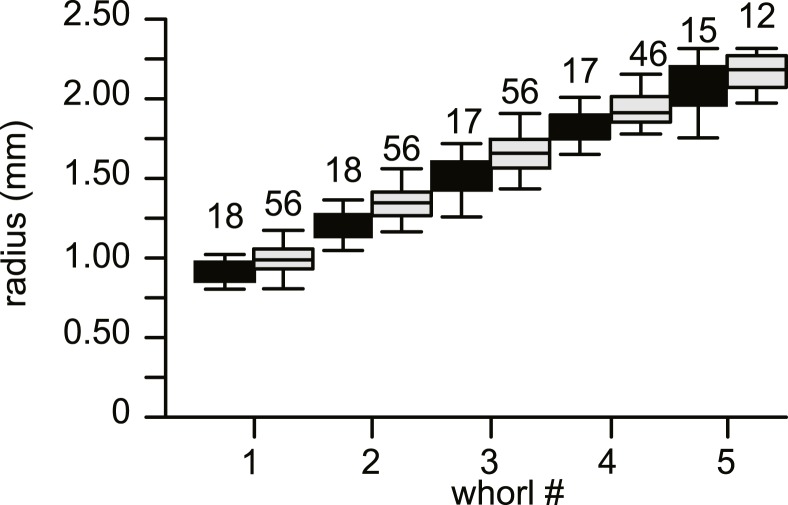
Average diameter of the whorls in 74 specimens of *Nummulites djokdjokartae*. Group A1 is indicated in black, group A2 in white. Error bars indicate 90th percentile. Numbers above error bars indicate number of specimens.

**Figure 5:**
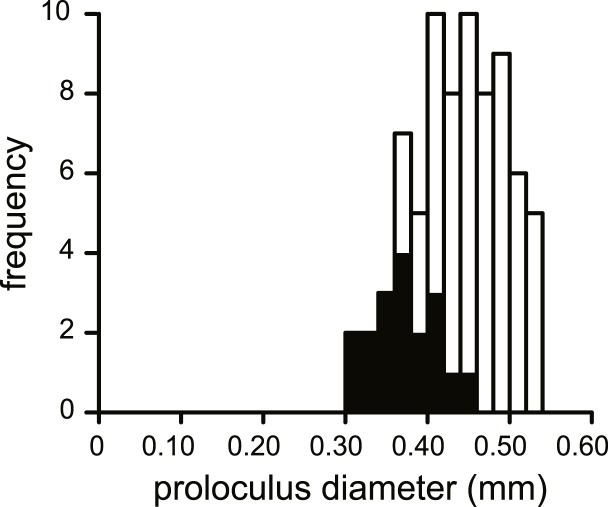
Histogram of the proloculus size in *N. djokdjokartae*. In black specimens with ≥5 whorls are indicated.

**Figure 6:**
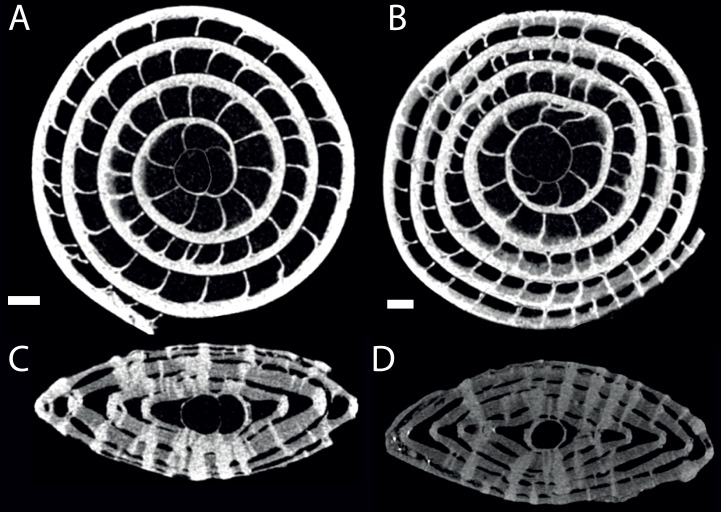
Virtual equatorial (A, B) and axial (C, D) sections of specimen 06KW01_02 (type A1; A, C) and 06KW0110 (type A2; B, D). Scale bars represent 0.5 mm.

**Figure 7:**
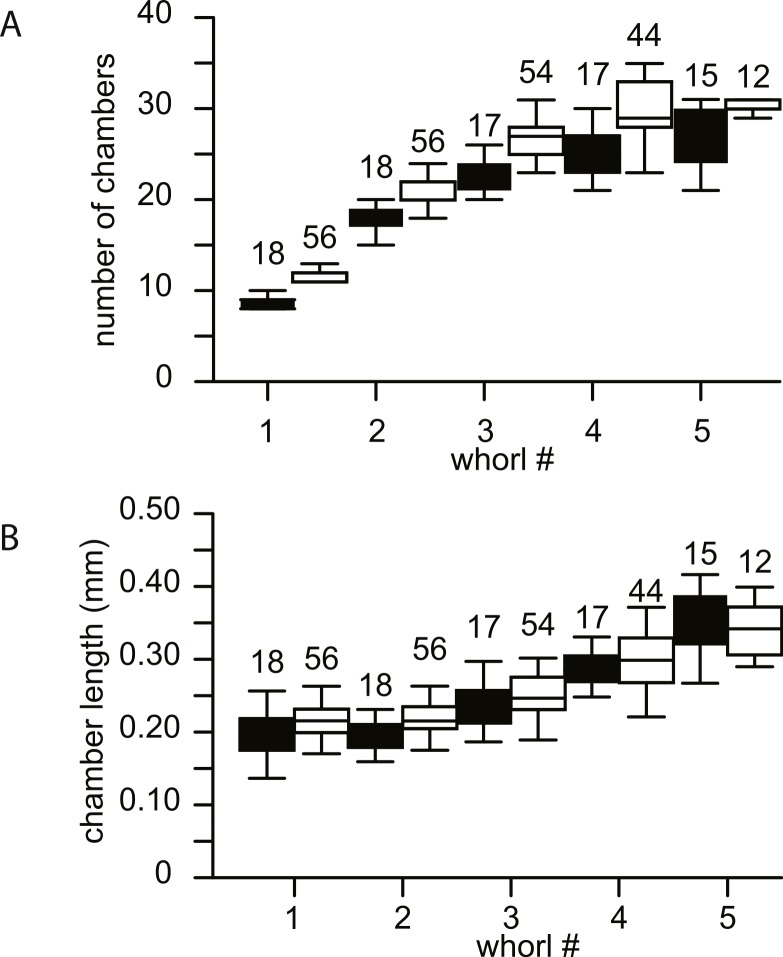
Biometrics of 74 specimens of *N. djokdjokartae*. (A) number of chambers per whorl; (B) chamber length. Group A1 is indicated in black, group A2 in white. Error bars indicate 90th percentile. Numbers above error bars indicate number of specimens.

**Figure 8:**
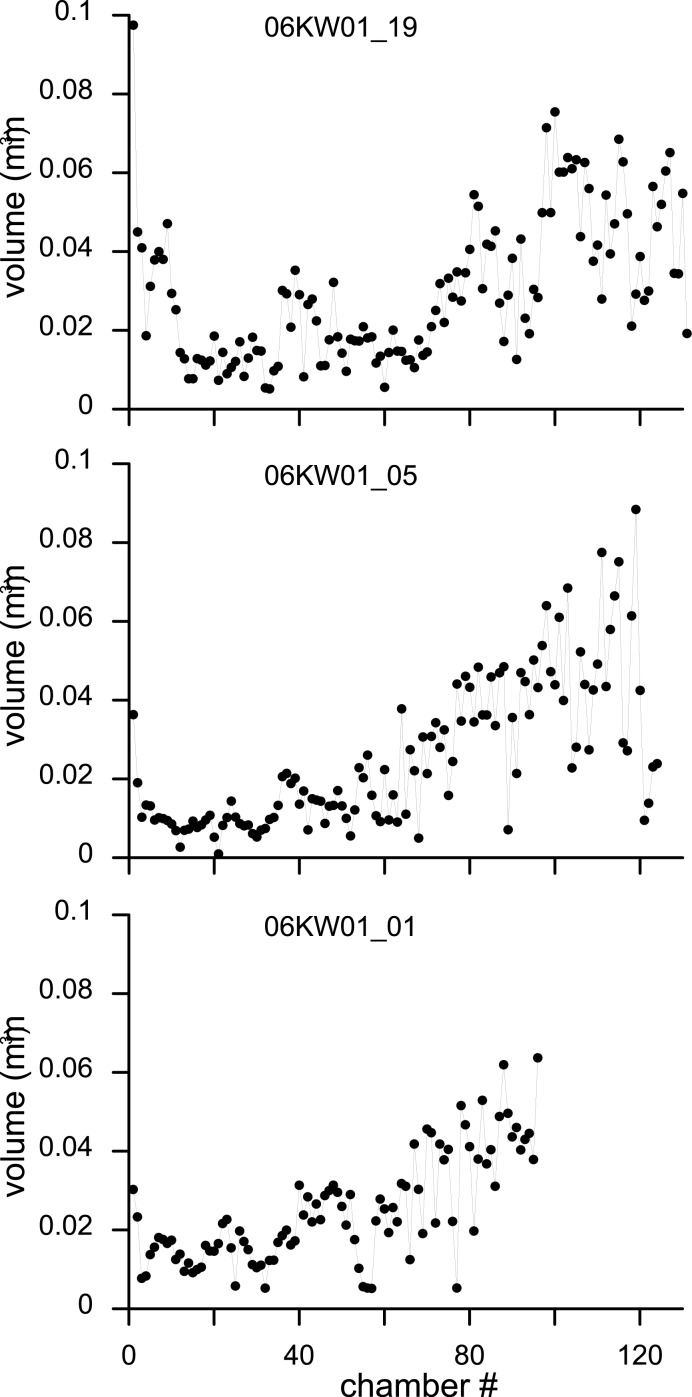
Variation in chamber volumes for three specimens of *N. djokdjokartae*. (A) specimen 06KW01_19 (shown in Fig. 3); (B) specimen 06KW01_05 (shown in Fig. 2); (C) specimen 06KW01_01.

Chamber shape in equatorial thin section has been related to chamber volume, potentially a more biologically relevant metric to estimate foraminiferal growth (Hohenegger & Briguglio, 2014). Chamber volume of the seven specimens segmented in this study shows a highly irregular pattern that does not compare to previous findings (e.g., Hohenegger & Briguglio, 2014; Briguglio, Hohenegger & Less, 2013). The initial 30–50 chambers hardly increase in volume, followed by a rapid increase in volume of the following chambers (Fig. 8). However in the latter phase occasional small chambers are also formed. These are probably the result of geometric constraints in chamber formation and serve to increase the regularity of the apertural face to facilitate growth in subsequent chambers. In the most comparable study relating growth increment to chamber volume in a complex nummulitids, i.e., the reticulate *Nummulites* species *N. fabianii* and *N. fichteli*, a pattern of stepped growth was identified (Briguglio, Hohenegger & Less, 2013). However, as is apparent in their figures, the alar prolongations, and therefore a substantial part of the chamber volume, were not included by these authors (Briguglio, Hohenegger & Less, 2013).

Chamber shape progressively becomes more complex, in the first whorl chambers are regularly involute with curved septal and septal filaments, followed by developing more irregular septal filaments, and extension of the alar prolongations. This comes together with increase asymmetry and chamber volume. A uniformitarian explanation of the biological function of our findings is not possible since comparable morphologies are not known in extant LBF (e.g., Hohenegger et al., 1999; Beavington-Penney & Racey, 2004; Renema, 2006). Possible explanations could include that the thin alar prolongations over the umbilical area allows for a more even distribution of symbiotic microalgae, while maintaining test strength. An alternative, but not exclusively so, explanation could be that this was an effective way of adjusting the diameter-thickness ratio in a variable environment. In extant LBF the diameter thickness ratio of the test is related to the hydrodynamic energy and depth (Hallock, 1979; Boltovskoy, Scott & Medioli, 1991; Mateu-Vicens, Pomar & Ferràndez-Cañadell, 2012). In shallow water tests are thick to prevent breakage and protect the symbionts from overexposure, whereas in deep environments the tests are flatter to increase the surface to volume ratio (Hallock, 1979). Variation in the extension of the alar prolongations over the central area of the test, such as observed for example between the specimens 06KW01_05 (Fig. 1) and 06KW01_19 (Fig. 3), could be a fast way of adjusting the diameter-thickness ratio during growth.

## Conclusion

We argue that to clarify uncertainties and improve the definitions of lineages within the genus *Nummulites*, three dimensional reconstructions of chamber shape and volume has the potential to provide important additional characters, next to equatorial and axial thin sections. Furthermore, these also provide insight into how long used characters, such as the shape of septal filaments can be related to chamber formation and, hence, growth.

## Supplemental Information

10.7717/peerj.1072/supp-1 Supplemental Information 1 Supplementary material with Fig. 2

10.7717/peerj.1072/supp-2 Supplemental Information 2 Supplementary material with Fig. 2A

10.7717/peerj.1072/supp-3 Supplemental Information 3 Supplementary material with Fig. 2B

10.7717/peerj.1072/supp-4 Supplemental Information 4 Supplementary material with Fig. 2C

10.7717/peerj.1072/supp-5 Supplemental Information 5 Supplementary material with Fig. 2D

10.7717/peerj.1072/supp-6 Supplemental Information 6 Supplementary material with Fig. 2E

10.7717/peerj.1072/supp-7 Supplemental Information 7 Supplementary material with Fig. 2F

10.7717/peerj.1072/supp-8 Supplemental Information 8 Supplementary material with Fig. 2G

10.7717/peerj.1072/supp-9 Movie S1 Supplementary material with Fig. 3A

10.7717/peerj.1072/supp-10 Movie S2 Supplementary material with Fig. 3B

10.7717/peerj.1072/supp-11 Movie S3 Supplementary material with Fig. 3C

10.7717/peerj.1072/supp-12 Movie S4 Supplementary material with Fig. 3D

10.7717/peerj.1072/supp-13 Movie S5 Supplementary material with Fig. 3E

10.7717/peerj.1072/supp-14 Movie S6 Supplementary material with Fig. 3F
